# Relationship between complement protein levels and tau pathology in pre‐symptomatic Alzheimer's disease resembles patterns observed in MCI

**DOI:** 10.1002/alz70856_104391

**Published:** 2025-12-24

**Authors:** Julia Loncke, Cynthia Picard, Henrik Zetterberg, Kaj Blennow, John C.S. C. S. Breitner, Judes Poirier, the PREVENT‐AD Research Group

**Affiliations:** ^1^ Integrated Program in Neuroscience, Faculty of Medicine and Health Science, McGill University, Montréal, QC, Canada; ^2^ Centre for Studies on Prevention of Alzheimer's Disease (StoP‐AD Centre), Douglas Mental Health University Institute, Montréal, QC, Canada; ^3^ Department of Psychiatry and Neurochemistry, Institute of Neuroscience and Physiology, the Sahlgrenska Academy at the University of Gothenburg, Mölndal, Sweden; ^4^ UK Dementia Research Institute at UCL, London, United Kingdom; ^5^ Clinical Neurochemistry Laboratory, Sahlgrenska University Hospital, Mölndal, Sweden; ^6^ Department of Psychiatry and Neurochemistry, University of Gothenburg, Mölndal, Sweden; ^7^ Department of Psychiatry, Faculty of Medicine, McGill University, Montréal, QC, Canada; ^8^ StoP‐AD Centre, Douglas Mental Health Institute Research Centre, Montréal, QC, Canada

## Abstract

**Background:**

The immune complement system mediates the physiologic pruning of superfluous or damaged synapses in the brain. Inappropriate hyperactivation of this complement system is believed partly to underlie neurodegenerative processes including Alzheimer's disease (AD) by contributing to excessive synapse loss, cerebral atrophy, and cognitive impairment. Elevated complement protein levels have been observed in the cerebrospinal fluid (CSF) of patients with confirmed AD; however, little is known about the role of complement in the pre‐symptomatic phase of AD when disease processes arise, as well as later AD stages.

**Methods:**

Baseline AD biomarkers Aβ42, pTau181, and total tau (Innogenetic ELISA) were contrasted with complement protein (C1q, C3, C3b, Factor H; Luminex) levels in CSF samples from 170 members of the PREVENT‐AD cohort with a parental or multiple‐sibling familial history of AD. Sex‐ and APOE4‐stratified analyzes were reproduced using SomaScan proteomic data from 708 cognitively normal, MCI, and AD subjects from the ADNI cohort. Complement levels were assessed using mass spectrometry and OLINK PEA technology and compared with synaptic markers (GAP43, SNAP25, SYT1, ADAM22, ADAM23) in a subset of PREVENT‐AD subjects. Cognitive functioning was assessed using total RBANS scores and subscales.

**Results:**

In PREVENT‐AD participants, pTau181 and total tau demonstrated strong and significant positive associations with C1q levels regardless of sex and APOE4 status, and with Factor H levels in women and APOE4 non‐carriers (all *p*‐values < .01). These associations more closely resemble the significant positive relationships of pTau181 and total tau with C1q among those with MCI and AD in the ADNI cohort versus those who are cognitively normal. C1q and Factor H also exhibited significant positive associations with synaptic proteins GAP 43, SNAP25, SYT1, and ADAM23 in PREVENT‐AD (all *p*‐values ≤ .01), but did not show association with cognitive performance.

**Conclusions:**

There is a clear relationship between complement levels, tau pathology, and synaptic integrity in asymptomatic individuals with familial history of AD. This closely mirrors the complement patterns observed in those with MCI and AD. Additional analysis of complement levels against volumetric MRI and PET imaging is ongoing.

